# Faculty Feedback Program Evaluation in CIMS Multan, Pakistan

**DOI:** 10.7759/cureus.8612

**Published:** 2020-06-14

**Authors:** Ambreen Shabbir, Hina Raja, Anjum A Qadri, Muhammad Hisaan Anjum Qadri

**Affiliations:** 1 Pathology, Combined Military Hospital (CMH) Institute of Medical Sciences, Multan, PAK; 2 Prosthodontics, CMH Lahore Medical College and Institute of Dentistry, Lahore, PAK; 3 Anaesthesiology, Combined Military Hospital (CMH) Institute of Medical Sciences, Multan, PAK; 4 Medicine, Al Nafees Medical College and Hospital, Islamabad, PAK

**Keywords:** faculty feedback, program evaluation, cims, multan

## Abstract

Faculty feedback program (FFP) at CMH Multan Institute of Medical Sciences (CIMS) was conducted for obtaining feedback for basic medical sciences faculty and evaluated to highlight its weaknesses for future improvement. The evaluation design was utilization-focused evaluation (UFE) keeping in mind its two essential elements. First element is the primary intended users (PIU) of the evaluation, namely the college faculty and students which were clearly identified and personally engaged to investigate intended use of the evaluation. Second element required the evaluator to ensure that the intended use of evaluation by PIU guide all other decisions made about the evaluation process. It was a mixed method study (qualitative and quantitative methods both) conducted from August 2018 to August 2019 in CIMS Multan with IRB approval following the steps of UFE. The whole program evaluation was conducted in two parts - first part constituted the 2018 manual FFP evaluation that provided suggestions for a FFP conducted in 2019 online. In step 2 the 2019 online FFP was evaluated again forming basis for future recommendations. Hence the PIUs response was recorded twice in the evaluation cycle - initially after the manual 2018 basic science FFP (response rate: 53%) - after which based on our findings a report was generated and recommendations suggested which were implemented in the 2019 online FFP and response observed again (response rate: 85.7%) to complete the evaluation cycle. Open-end questions were asked from faculty (qualitative analysis) with three themes emerging regarding FFP procedure, questionnaire and timing. An acknowledgement of shift of FFP procedure from manual (2018) to online system (2019) was observed in which faculty praised the ease (72.2%), confidentiality (66.6%), anonymity (50%) and transparency (33.3%) of the online system compared to manual FFP, which was reported to be a rather tense experience (83%). Regarding questionnaire, 38% faculty members reported feedback questions asked from students to be vague and 66.6% claimed that the timing was inappropriate and should have been end of academic year. When asked for suggestions for improvement in 2018 FFP, 72% faculty suggested training students on providing feedback and making the procedure user friendly (83%). Student response regarding both feedback was obtained online by a survey with closed ended questions (quantitative study). Fifty-three percent college students were satisfied with the online FFP giving an average rating of 3.2 to the software user interface and 85% affirmed that using the online software aided in providing anonymous responses helping them provide candid feedback. Seventy-five percent students agreed that online feedback system in 2019 had streamlined the feedback process and made it more efficient compared to the paper-based manual survey of 2018. After evaluating 2019 online FFP, few suggestions were recommended for future FFP including obtaining formative as well as summative faculty feedback, supplementing feedback with teacher’s self-assessment/pen picture and incorporating 360 multi-source feedback.

## Introduction

Program evaluation is a systematic collection and analysis of information regarding a broad range of topics related to the design, process, implementation, and outcomes of a program to monitor and improve its quality and effectiveness [1,2]. Any kind of educational program is intended to bring about change and program evaluation investigates whether this change (intended or unintended) has occurred or not [3]. In the dynamic environment of medical education, innovations like virtual reality and advanced simulations etc. have drastically changed the teaching/learning methods for medical students. When these innovations are incorporated into teaching methodologies, medical educationists need to evaluate whether they significantly enhance student learning with a cost effective benefit or not [4]. Several designs for program evaluation exist. One of them is a classic model which either focuses on the objective of the program, process or the outcome. The second is utilisation-focused which best suits health care programs as it is a flexible model that can be moulded according to aim of program evaluation [4-7].

Feedback is the constructive yet objective appraisal of performance provided for the purpose of improving skills [8]. Along with being a fundamental component of medical education ensuring quality and promoting learning it is the most difficult aspect too [9]. Evaluating effectiveness of teaching by learners through structured questionnaires is a common practice in developed countries [10]. It can either be formative or summative depending upon the intention and time at which it is taken with the purpose of setting standards for function, performance and structure of medical schools [11]. Feedback consists of communication of information followed by reactions to such communication. In medical education, it is a significant element of teaching/learning by encouraging and enhancing the learners' knowledge, skills, attitude and performance thus helping them reach their goals [12]. In the absence of feedback learners rely on self-assessment to evaluate what went well and what demands improvement, which does not fully identify learners' own strengths or weaknesses. Learners may interpret absence of feedback as approval of their performance, which may not be the case. Feedback can only be utilised to its maximum potential when learner is receptive to suggestions and willing to improve. Thus not only the feedback questionnaires but the entire feedback procedure needs to be user friendly [11].

The faculty feedback program (FFP) at CMH Multan Institute of Medical Sciences (CIMS) was tested for obtaining feedback for basic medical sciences faculty and evaluated to highlight areas for future improvement. The primary concern of administration was to address the faculty objections regarding procedure, questionnaire and timing of FFP, which warranted an evaluation. To conduct a thorough evaluation, the program had to be studied in detail to observe if the goals, objectives and outcomes of the program were aligned [5].

## Materials and methods

We followed utilization-focused evaluation (UFE) approach as it stresses that evaluations are to be judged by their utility/use, evaluators are facilitators of the evaluation process who design it considering how the process of program affects its use and the focus is on intended use by primary intended users (PIU) [13]. The flexible model of UFE suited our purpose as the evaluators could develop a working relationship with PIU helping them determine the kind of evaluation they required [13]. The PIU in our research were the full-time basic science faculty (34 faculty members whose feedback was being obtained about their teaching) and medical college students of 1st, 2nd, 3rd, 4th and 5th years (who gave the feedback). Evaluation was conducted in CIMS Multan from August 2018 to August 2019 with IRB approval. PIU response was recorded twice in the evaluation cycle - initially after the 2018 manual FFP - after which an evaluation report was generated based on our findings and recommendations suggested which were implemented in the 2019 online FFP and response observed again for continued improvement.

It was a mixed method study with evaluation team comprising of internal evaluators from department of basic sciences selected on basis of their qualification in health professional’s education working in close coordination with department of medical education. FFP evaluation was carried out by addressing all the steps of UFE (Table 1) [6].

**Table 1:** UFE steps in a nutshell PIU: Primary intended users; UFE: Utilization-focused evaluation.

Assess institute and evaluators readiness
Identify/Engage PIU and conduct situational analysis with them
Identify primary use and consider and build in process uses
Focus priority evaluation questions and check if evaluation inquiry fundamental areas are being addressed
Determine intervention model/theory of change being evaluated as well as appropriate methods to generate credible findings while supporting intended use by PIU
Brief PIU of controversies about methods/their implications. And simulate use of findings.
Gather data with attention to use and organize/present data for use by PIU
Prepare an evaluation report/disseminate significant findings and follow up with PIUs to enhance use.
Meta evaluation of use

The process of evaluation was built considering primary goal of FFP and procedure being followed with its limitations, concerns raised by PIUs along with their feedback which helped in formulation of statement of evaluation questions (Table 2).

**Table 2:** Statement of evaluation questions

Does faculty feedback program address any significant issue/problem related to medical education?
Does it constitute well-defined goals and objectives?
What is the source of funding of this program and is it sufficient to meet its needs?
Do the participants of this program understand the significance of this program?
Have concerns of all stakeholders been addressed in the planning of feedback program?
Was the program online or manual?
What were the tools used for data collection in the program?
Were the tools utilized for obtaining feedback valid?
Have the participants been briefed and trained regarding the working of feedback program?
Which department has been assigned the duty of receiving feedback?
How has confidentiality been ensured on the feedback system?
Who shall have access to the feedback?
Have policies been developed for training of individuals/groups based on their feedback?
Is the faculty development committee of medical education on board?
Who has been authorized to conduct counseling sessions for individuals?
How will the feedback program be evaluated regularly?
How long has the program been running and during that time has the program progress been reviewed?
How frequently is the auditing of feedback program carried out?

To assess and enhance readiness for evaluation, meetings were scheduled between the basic science faculty, student representatives, college administration, department of medical education, IT and software experts. PIU concerns were shared and minutes of the meeting recorded. The 2018 FFP was a manual system comprising of a feedback form that was distributed to students after their learning session in the lecture hall. Once feedback was obtained, it was analyzed manually and a report generated for concerned teacher regarding their teaching which was handed over to them with caution advised to those who received negative feedback. To acquire a written response of faculty regarding this system, a form was circulated to be filled manually by them with three open-ended questions about strengths and weaknesses of FFP and suggestions for improvement. The same response form was used to obtain their comments after the 2019 FFP. To obtain students' views regarding FFP, a self-administrative online questionnaire (via survey monkey) was sent to them with closed-ended questions regarding their preference between the manual FFP (2018) and the online one (2019) and views about the online software (alphaxlibrary) used to obtain the 2019 online feedback.

Additional method of data collection included direct observation of FFP implementation, minutes of the meeting, document review of feedback form used in the FFP, data generated from the FFP and review of feedback questionnaires/form (included articles to determine a valid questionnaire) to provide guideline from Nishter Medical College [14], Agha Khan Medical College [10], Bahria University Medical and Dental College [15].

## Results

After the manual FFP, the response rate of faculty to our evaluation form for FFP was 53%. Once this evaluation was completed, recommendations suggested and implemented in 2019, we acquired its feedback from faculty to evaluate the new program and received an increased response rate of 85.7%. In both instances, faculty concerns revolved around three main themes - FFP procedure, timing and questionnaire used in the program (Table 3).

**Table 3:** Faculty responses to manual FFP (2018) and online FFP (2019) FFP: Faculty feedback program

Aspects	Themes	Percentage of participants with different responses to manual 2018 FFP (%age)	Percentage of participants with different responses to the online 2019 FFP (%age)
		Strengths	Procedure	Good Initiative - 16.6	Good Online system - 66.6
				Helps Improve Teaching = 50	Anonymous = 50
	Confidential = 38.8		
	Transparent = 33.3		
	Easy Method = 72.2		
	Easy to Understand Results = 33.3		
	Good Feedback Delivery = 11		
	Helps Improve Teaching = 33.3		
	Good Software = 27.7		
	Gives Insight to How Our Teaching is Assessed = 5		
Questionnaire	Addressed Teacher’s Knowledge and Attitude = 16.6		Addressed All Teaching Aspects = 22.2
			Identifies Teacher’s Strengths and Weaknesses = 22.2
			Questions Were Relevant = 5
			Questions Were Adequate = 5
Timing	None		None
Weaknesses	Procedure	Lacked professionalism = 38.8	Students Were Untrained = 27.7
		Lacked Confidentiality = 5	Students Showed Bias = 33.3
		Was Manual (Not Online) = 11	Unclear Pie Charts = 5
		Resulted in faculty counselling = 38.8	Results Not Explained = 5
		Students Showed Bias = 44.4	Results Difficult to Comprehend = 11
			Small Sample Size = 16.6
	Questionnaire	Same Questionnaire for All Types of Teaching Sessions = 5	Same Questionnaire for All Types of Teaching Sessions = 11
		Questions Were Unclear = 38.8	Required Detailed Suggestions by Students to Teachers = 11
			Needs Detailed Questions = 5
			Requires Teacher’s Pen Picture = 5
	Timing	Feedback Timing Was Inappropriate (in mid of academic year) = 66.6	Timing was inappropriate = 11
		Students Were Not Given Ample Time To Fill The Form = 44.4	
Suggestions for Improvement	Procedure	Feedback Should Be Taken from Good Students Only = 16.6	Design 360 Feedback = 22.2
		Train Students = 72.2	Take Two Directional Feedback = 22.2
		Train Faculty = 5	Train Students = 11
		Take Large Sample = 5	Aim to Improve Not Defame = 5
		Feedback Procedure Should Be Friendly/Non-Humiliating = 83.3	Try Feedback Delivery Online = 11
		Should Be Online = 27.7	Should Be Used Constructively = 11
		Should Be Confidential = 27.7	Prevent Student Bias = 5
		Should Not Have Student Bias = 33.3	Include Whole Class = 5
		Should Be 360 = 5	Include Previous Class Too = 5
		Should Be Peer Feedback = 11	Compare Teacher Performance Regularly = 5
		Should Be Two Way Feedback = 5	Reward Teacher With Good Feedback = 5
		Should Give Statistics = 5	Results Should Have A Concluding Statement = 5
		Should:	Should Have Different Questionnaire for Different Teacher Levels = 5
	Questionnaire	Be Separate for Junior and Senior Faculty = 5	Questions Were Not Proper = 5
		Be Separate for Large and Small Group Discussion = 5	Add More Open-Ended Qs = 5
		Make Questionnaire Acc. To Subject = 5	Add More Weightage to Written Open Comments = 5
		Use Easy Questions = 38.8	Should Have Qs To Evaluate Teaching Environment Not Teacher = 5
		Use Open-Ended Questions = 5	
		Should:	Should Be taken:
	Timing	Be at End of Year = 16.6	Annually = 5
		Be at Midterm = 5	Biannually = 11
		Give Teacher Time to Improve Before Next Feedback = 5	Regularly = 27.7
		Give Student Time To Fill Form = 33.3	

Regarding manual FFP procedure, a basic science teacher suggested, “the feedback system should be friendly and students trained to provide feedback of their teachers” while another commented, “the procedure of feedback delivery should be confidential and anonymous”. One faculty member suggested, “faculty feedback should be delivered to the teachers in an encouraging manner”.

About the questionnaire, the faculty felt “the wording of feedback questionnaire should be very simple and clear for the students”. Faculty had various views about feedback timing and one member said “feedback should have been taken not at the start of academic year but at the end of the year to avoid favouritism and bias” while another recommended “feedback should be taken at least three months after the academic year has started and students should be given ample time to fill out the forms”.

Comparison of basic science faculty comments on FFP of 2018 and 2019 is provided in Table 3 with an acknowledgement of shift of FFP from manual (2018) to online system (2019) praising the ease (72.2%), confidentiality (66.6%), anonymity (50%) and transparency (33.3%) of the online system. While 2018 manual FFP was considered unprofessional by 83.3% of faculty with 38% reporting vague feedback questions being asked from students, the 2019 online FFP was praised for its procedure of receiving feedback and delivering results to faculty in the form of stats and pie charts, all conducted confidentially. The 2019 online questionnaire was appreciated by 22.2% teachers reporting the questions being relevant addressing all teaching aspects. Having said that 27.7% still believed after the online feedback that students were untrained in providing feedback (27.7%) and showed bias (33.3%). When asked for suggestions for improvement in 2018 FFP, 72% faculty suggested training students on providing feedback and making the procedure user friendly (83%).

One basic science faculty member commented “the 2019 FFP was online, computerised and accurate, convenient for students to attempt and provided us with graphical results which were easy to interpret but students still lacked training in giving feedback and they must be made to realise they are not RATING teachers” and about questionnaire he stated “the feedback form needed to address teachers for small and large group discussions separately” and on timing it was suggested “it should be taken at end of academic session and not at the start”.

Another faculty member observed “the software used for feedback seemed appropriate as it provided immediate response as opposed to the paper-based survey of 2018 but students still need to be trained on the software and I believe the teacher’s pen picture should also be added in the feedback program”.

Among all medical years which were included in both the 2018’s manual and 2019’s online FFP, 53% students were satisfied with the online FFP while 11% were dissatisfied and 36% were neither. Students response regarding both feedback was obtained online by a survey with closed-ended questions. They gave an average rating of 3.2 out of 5 to the software user interface and suggested they required more training for it. 85% students affirmed that using the online software aided in providing anonymous responses and helped them in giving candid feedback while 75% agreed (23% strongly agreed and 52% agreed) that online feedback system in 2019 had streamlined the feedback process and made it more efficient compared to the paper-based manual survey of 2018. In open comments, one student mentioned of having log in issues (students were provided Log in ID and passwords), while another presumed that the method would take some time to establish and improve.

## Discussion

Medical institutes must sustain a high education standard as their graduates carry great responsibilities for which purpose evaluations are carried out to equate control with accountability and quality enhancement [16-20]. Institutes use systems to provide feedback to their teachers in order to improve their performance and frequent written feedback is one way to increase teacher evaluation scores, but despite an overall encouraging attitude towards evaluations, it has been observed that use of evaluation data for teaching improvement is less [8,21]. Evidence suggests that student satisfaction is closely related to tangible action taken after they provide teacher feedback [22]. Several factors play a role in success of teacher feedback programs like characteristics of feedback provider and recipients, its procedure and timing as well as its content. Ignoring these factors can lead to detrimental effects on teachers/students and their performance [23]. To look out for such negative effects, faculty feedback program evaluation was conducted in CIMS. Not only was the program evaluated once in 2018 but also a second time in 2019 to gauge the student/teacher response and satisfaction to the suggested changes in evaluation report.

The 2018 FFP evaluation report stated that although the FFP had clearly a demarcated goal of teaching improvement by acquiring insight to faculty teaching including their strengths and weaknesses, but the objectives needed definition. Before the start of program, a detailed briefing regarding goals, objective and expected outcomes of the program to stakeholders was lacking. The feedback tool used was a paper-based survey with closed-ended leading questions circulated to be filled by students in lecture halls in the presence of teachers, which could increase anxiety levels of students [24]. Although students were briefed about the feedback form, many revealed they could not clearly understand the survey questions. Protocols to deliver feedback results to teachers needed to be refined. Based on the data collected after 2018 manual FFP, suggestions were recommended, disseminated and implemented in the 2019 FFP. The new-implemented guidelines were focused on a shift from manual to online feedback system [25]. For this purpose, feedback software was developed in a learning management system (alphaxlibrary). Feedback was collected by providing students their login credentials allowing them to submit feedback anonymously. The issue of student training was resolved by coaching them on the software to ascertain ease of feedback provision in an appropriate environment with ample time. The software could perform analysis and present results in graphs/pie charts making it convenient to analyse feedback. Feedback survey form was modified and divided in two sections - first section had closed-ended statements about the teacher’s teaching/organising skills. It was scored on a Likert scale. At the end of this section space was provided for comments regarding teacher’s strengths, weaknesses and suggestions for improvement in that domain. The second section addressed the teacher’s interpersonal skills with closed-ended statements and open comments in the same format as above. This aided in acquiring quantitative as well as qualitative data regarding teaching. A written summary of teacher’s online feedback in sealed envelope was provided to them individually in private ensuring confidentiality. Tailoring of teaching strategies was advised according to the feedback and it was suggested that teacher feedback be used to provide needs assessment for faculty development workshops.

In order to complete the evaluation cycle, the new 2019 online FFP was evaluated as well. It formed basis for future recommendations and was according to PIUs suggestions. Supplementing feedback with a pen picture/self-assessment of the teacher was advised as it aids in prompt acceptance of student feedback when the teacher has performed self-analysis honestly [16]. It was also advocated that summative as well as formative feedback be acquired. Formative feedback helps instructor/learner to improve for next module, summative could guide their work technique and attitude for the next academic year [17]. FFP should incorporate 360 multi-source feedback as students are not always the best judge to evaluate teacher’s methodology and this alone can lead to bias [16,18].

A similar UFE model was used at Bahria University Medical & Dental College (BUMDC) Pakistan to evaluate integrated learning program (ILP) of neurosciences. The evaluators engaged PIUs, formulated the key evaluation questions after listening to their concerns and followed UFE stepwise as mentioned in Table 1. For data collection, they conducted three focused group discussions (FGDs) with PIUs. They also analysed results of nonintegrated and integrated module after ILP, guide book for students, schedule of both modules, feedback forms filled by students and faculty members [16]. Our evaluation did not have FGDs but we were able to collect qualitative data from students via acquiring their comments on teacher’s strengths, weaknesses and suggestions for improvement. We recommend dialogue-based survey in the future, which is a superior alternative to quantitative methods possessing potential to facilitate reflection/dialogue between students and teacher about their learning. Little research exists on qualitative evaluation methods compared to quantitative ones [26]. But in a study published in January 2020, it was observed that open-ended questions providing space for explanation of low teachers ratings were considered important by students specially in questionnaires having non-specific questions [21]. We had modified the 2018 feedback questionnaire to target specific qualities, e.g., time management, command on subject, outlining learning outcomes, conduction of interactive sessions in a safe learning environment, use of real-life experiences, multiple teaching aids and innovative teaching methods, etc. It additionally addressed teacher’s communication/mentoring skills and accommodated open comments by students. However, even after revision/modification of the questionnaire, our focus remained teacher oriented while a learning-oriented approach is recommended in the future [21].

For obtaining teacher’s opinion about the feedback system, we used a qualitative approach, so a candid response can be acquired on their behalf. While this method supplied us qualitative data, it is argued in contrast to surveys, dialogues invite students/teachers to provide feedback about aspects they regarded relevant to their learning/teaching processes thus relating to their expected use of evaluation [21]. In UFE, evaluation is judged by its utility for its PIUs. The intended use of evaluation was improved teaching and learning with PIU as teachers and students. Involvement of PIU can occur in the planning stage of an evaluation as was witnessed in our evaluation for generating evaluation questions, but an active involvement can also be in phase of analysis or implementation of findings, which was lacking in our evaluation [27]. We recommend involving PIUs in this part of evaluation as well for better use of evaluation report and data [21].

## Conclusions

Evaluation of educational programs demands a skill set that enables the evaluator to assess multiple variables, which may affect the outcomes of a given educational program and hence requires evaluator expertise. At the same time in faculty feedback programs difference between faculty and student perception does tend to emerge but by using the UFE, evaluators can actively engage them in formulating recommendations for improvement. The limitations in this process was the time period taken for application of new FFP and its evaluation to complete the evaluation cycle. Also, the faculty demanded separate questionnaires/feedback survey forms for large interactive group sessions, small group case-based learning and practical sessions which could not be implemented as developing a valid survey form for each was beyond the scope of our study and demanded another set of expertise.
